# Differentiation between Chancre and Chancroid

**Published:** 1886-01

**Authors:** T. J. Bennett

**Affiliations:** Austin, Texas


### DIFFERENTIATION BETWEEN CHANCRE AND CHANCROID, WITH TABLE.

*By T. J. Bennett, M. D., Austin, Texas.*

Read before Travis County Medical Society, and by vote referred for publication to Daniel's Texas Medical Journal.

THE literature upon this subject is not at all uniform as to the terms employed to distinguish the two forms of disease. According to some, the term *syphilis* means both the local and constitutional affection; while others seem to make a distinction. Yet, the word *chancre* is used indiscriminately to express the sore in both diseases, while there are still others who teach that syphilis means the constitutional form only, and chancre is the term which expresses this form, and that *chancroid* is entirely distinct and expresses only the local disorder.

This latter view is the one adopted in this paper, and from all that can be gathered upon the subject, is as much a demonstrated fact today as, perhaps, any point in the science of disease, and requires only constitutional treatment; while the chancroid is distinctly a local disease and requires only local treatment.

We have only to glance at the constitutional symptoms without entering into details to see the importance of a clear differentiation. Three-fourths of all aneurisms, apoplexias, and perhaps a still larger per cent of all diathetic diseases are traceable to this constitutional poison.

With the lights before us as to symptomatology, pathology and etiology, ignorance upon the subject seems inexcusable. To drop into ruts and prescribe a routine of mercury and caustics on "general principles," seems to be due more to carelessness than to any other cause.

The privacy of the disease, its filthiness, as well as the low class of society represented by its victims, together with a correspondingly low and uncertain compensation for services, no doubt, go to make up some of the excuses for neglecting this branch of practice. There are few of us who have not had patients come in for treatment presenting all the symptoms of unmistakable secondary syphilis and with the glans penis or prepuce terribly burned up by the use of caustics; while on the other hand, we have seen these unfortunates ptyalized almost incurably with nothing to show for it but a soft suppurating local sore on the penis. It is true, the druggists are accused of a great deal of this kind of practice, but it must not be understood that they alone are responsible.

The question is: How are these diseases to be distinguished?

Contamination with these poisons is brought about by the same process—by the poison coming in contact with an abraded mucous surface. This is the cause for the most part, though it may be introduced through a broken surface anywhere, and syphilis by heredity.

Three or four weeks elapse before the constitutional poison manifests itself, which is always at the point of inoculation in the form of a papule. This papule appears to "extend laterally, and in eight or ten days becomes scaly or breaks down into an ulcer, secreting a very small quantity of serous or puriform liquid, which presently dries up into a crust or scab." The base becomes indurated and disc-like, yielding to the touch a button-like feel beneath the integument. This induration, according to Zeigler, is due to an "extreme cellular infiltration of the integument, without any very special histological features." The lesions which distinguish syphilis from other diseases more especially, are, according to T. Henry Green, of London, "certain fibroid indurations," as the initial sclerosis described above, a " form of growth known as *jumma* or syphilitic tumor, and certain changes in the arteries." The development of this fibroid tissue, however, is not unlike that which is met with as the result of ordinary chronic inflammation in common connective tissue. But the peculiarities which are so characteristic of syphilis consist in their distribution and localization. "They occupy for the most part *small areas* and the *surrounding tissues are unaffected.*"

But it is not the purpose of this paper to extend investigation into the pathological and histological differences between syphilis and other constitutional diseases, but more especially to differentiate clinically, pathologically, and otherwise, as well as to group in a tangible form, all of the prominent differences between the constitutional and local sores of the two diseases under consideration.

The chancroid, like the chancre, is a contagious venereal affection, but the period of incubation, instead of being three or four weeks, is from a few hours to three or fourdays. The sore appears, like the chancre, at the point of incuba tion, but in the form of a vesicle or pustule, and itrapidly becomes an ulcer with a yellowish soft base, instead of a papule with a dry brownish hard base. "It grows by a progressive molecular death of border-tissue," the edges and base at first becoming "slightly infiltrated with cells, and these as they near the surface pass through successive stages of degeneration and decay, and at length form a layer of structureless detritus."

Cornil, of Paris, taught in 1882, that there was no special difference between the cellular infiltrations in the two sores, nor for that matter did they "differ from the cell growth in other granulomatous ulcers—notably in tuberculous affections," but that the tendency in the chancre was "to persist unchanged for a considerable time while in the chancroid they very speedily break down." This difference, of course, like the difference existing between any other two dissimilar diseases is due to the difference in the nature and character of the two poisons producing them.

These poisons are supposed to be forms of living organisms, but at the present state of knowledge upon the subject, like many others of the so-called germ diseases, remains a mere supposition, which it is not necessary to discuss here.

Bryant teaches that we cannot tell with anything like certainty whether a sore is going to be local or constitutional. He is inclined to think the poison is identical, and that the peculiar idiosyncrasy of the patient nearly or entirely controls the course the disease will take. He believes that the sloughing ulcerative process in the chancroid prevents absorption of virus which would otherwise produce the constitutional disorder. All of this seems too hypothetical.

The sore in syphilis is always single, while in chancroid it is single or multiple. The ulceration is always passive in the chancre, except when the original character of the sore has been changed by the use of caustics or other agents, while in chancroid it is invariably active. Another striking difference is manifested in the spread of the chancroid poison, forming other similar sores wherever it comes in contact with an abraded surface, thus becoming inoculable to an indefinite extent, while the secretion from the chancre never causes another similar sore, nor is ever inoculable a second time in the same person, nor in the lower animals, as the chancroid poison is. Also the glandular system exhibits a marked difference. In the chancre the inguinal glands enlarge and become hard, forming a row of four or five on either side, but are not tender to touch, nor do they ever suppurate, except when bruised or in a highly cachectic subject, for it is a fact well established that inflammatory processes in the syphilitic are set up with the same facility that a stubble would be caught on fire by a torch thrust into it. Not only do these glands enlarge, but the cervical, axillary, and others glands of the body. In the chancroid the bubo is generally single, tender to press upon, and seldom fails to suppurate. The other glands are never affected. Still another feature is the treatment. The chancre never yields to local treatment; in fact local treatment generally aggravates the sore, but to constitutional treatment only, and requires from six months to one or two years for cure, while the chancroid yields to local treatment altogether, and requires but ten to twenty days to effect a cure.

As to treatment in either form, I have nothing special to offer that cannot be found in the late text books, except that I may mention saccharated pepsin as a local remedy in mild forms of chancroid. It has given good results in the few cases in which I have employed it, seeming to prevent further ulcerative action, and to cut short the disease in four or five days. I order it dusted over the sore plentifully, three or four times a day. How it acts, by what *modus operandi*, I am unable to say. Whether by a digestive process upon the virus, thereby rendering it inert, so far as further destructive action is concerned, or whether it is the sugar with its reputed antiseptic and plastic properties. I have been sufficiently encouraged, however, to suggest a further trial of this new agent.

In conclusion, and by way of summary I present herewith a table giving in contrast the most prominent and reliable symptoms of chancre and chancroid:

| CHANCRE. | CHANCROID. |
|---|---|
| 1. Period of incubation 18 to 90 days. | 1. A few hours to 3 or 4 days. |
| 2. Appears in form of papule. | 2. Appears in form of vesicle or pustule. |
| 3. Cellular infiltration extends laterally, and does not break down. | 3. Extends laterally and breaks down by molecular death. |
| 4. Sore, hard and dry. | 4. Sore, soft and suppurating. |
| 5. Ulcerative action always passive. | 5. Ulcerative action always active. |
| 6. Never spreads. | 6. May spread indefinitely. |
| 7. Never inoculable in same person nor in lower animals. | 7. Inoculable indefinitely in same persons and in lower animals. |
| 8. Buboes always multiple and seldom suppurate. | 8. Seldom more than one and nearly always suppurate. |
| 9. Cervical and axillary glands always enlarged. | 9. Never enlarged. |

| | |
|---|---|
| 10. Yields to constitutional treatment only. | 10. Yields to local treatment only. |
| 11. Six months to two years treatment required. | 11. Fifteen to twenty days treatment required. |

It will be understood that each of the eleven differences enumerated above is subject to slight exception ; also it is to be borne in mind that only typical cases of the two diseases are outlined, and that the complications which seem so apparent are to a greater or less extent subjected to the above differentiations, and that, further, to every physician in the diagnosis must come the conviction that it is plainly a case of *no medicine* vs. *a grtat deal of medicine*, and as to prognosis *no danger* vs. *a great deal of danger.*

